# An On-Device Edge AI Agent for Reference-Free Self-Diagnosis of Low-Cost Multi-Pollutant Sensors

**DOI:** 10.3390/s26144526

**Published:** 2026-07-16

**Authors:** Yinan Wang, Tianqi Wang, Yubing Pan

**Affiliations:** 1 Key Laboratory of Middle Atmosphere and Global Environment Observation (LAGEO), Institute of Atmospheric Physics, Chinese Academy of Sciences, Beijing 100029, China; 2 College of Electronic Engineering, Chengdu University of Information Technology, Chengdu 610225, China; 3240308008@stu.cuit.edu.cn; 3 Institute of Urban Meteorology, China Meteorological Administration (CMA), Beijing 100089, China

**Keywords:** personal exposure monitoring, indoor air quality, edge AI agent, reference-free sensor self-diagnosis, low-cost air-quality sensors, cross-interference, on-device reasoning, skill-based sensor management

## Abstract

Low-cost multi-pollutant sensors make personal exposure monitoring affordable, but assuring their data quality in the field is the bottleneck, while current devices leave it to remote servers: the field unit is a passive terminal that cannot self-check its sensors, takes days to accept a new one, and loses quality control whenever connectivity drops. We develop Zhiwei, an on-device edge AI agent for personal exposure monitoring that brings the reasoning loop onto the device, so it can diagnose its own sensors without a reference, onboard new ones through a declarative skill package with a capability-association graph, and keep working offline through a three-tier cloud-to-rule-engine fallback. We validate these capabilities, rather than field exposure tracking, in a 30-day fixed indoor deployment in Beijing of 1,896,789 records at 99.9% completeness. The agent decided on its own, without a reference, which channels to trust, identifying that the nominal ozone channel measures total oxidizing gas rather than ozone alone, a conclusion the manufacturer’s datasheet independently confirms, while the PM_2.5_ and NO_2_ channels were separately corroborated as relatively usable against a nearby station (r = 0.90 and 0.86). Under a simulated cloud outage, it kept data collection uninterrupted by handing inference to the on-device local model. This is a single fixed indoor site and a design-and-functional validation; evaluation under mobile, rapidly changing microenvironments is future field work. Zhiwei shows that an environmental sensing device can manage its own data quality autonomously on-device, a prerequisite for trustworthy personal exposure monitoring.

## 1. Introduction

Air pollution causes approximately seven million premature deaths each year [1], and long-term exposure to fine particulate matter is an established, independent risk factor for cardiovascular disease, respiratory disease, and lung cancer [2,3]. Because health risk is governed by what a person actually breathes rather than by the regional average reported at a fixed station, environmental health research increasingly seeks to quantify exposure for the individual [4,5]. Personal environmental exposure monitoring answers this need by carrying multi-pollutant sensors with the individual, with the long-term aim of recording exposure along real-life trajectories at high temporal resolution. Advances in low-cost electrochemical and optical sensors have made this practical: portable instruments now report on the order of eleven pollutant parameters, from the synchronous gas and size-resolved particulate monitoring of the SEARCH platform [6] to pen-sized and wearable form factors validated in the field [7,8,9], giving the discipline the hardware basis for high-resolution individual-exposure cohorts [10,11,12].

This progress in hardware has not been matched in data-quality assurance, for an architectural reason. Most current devices make the same choice: the perceptual intelligence lives on remote servers, while the field device is a passive terminal that collects and uploads. The most immediate consequence is that a sensor cannot recognize when it has gone wrong. Electrochemical gas sensors drift at zero, cross-interfere, and lose sensitivity with age [10], and the standard countermeasures all leave the device unaware of its own state: factory calibration decays once the instrument leaves the laboratory [13]; field and machine-learning calibration against a co-located reference captures only the moment of calibration and presupposes the very reference that field deployments lack [14,15,16,17,18]; drift-compensation methods run offline on archived data [19,20,21]; and server-side anomaly detection reacts only hours or days later. Throughout, the device keeps producing data of unknown quality and uploading them for later judgment [11].

The same remoteness makes the device rigid and fragile. Adding a sensor for a newly relevant pollutant or an unexpected event means rewriting drivers, retraining calibration, updating the reasoning logic, and redeploying the stack, a cycle of days that freezes the device’s perceptual boundary just when flexibility is most needed. Fragility appears whenever connectivity is lost, which over field 4G or 5G links is routine: an outage strips every quality-assurance function at once, so readings gathered while the link is down are uploaded later but can no longer be traced to a known-good state. For the remote-region and high-density cohorts that motivate the field, this silent loss of control threatens the validity of an entire study.

Efforts to move intelligence toward the device have so far stopped short of what self-management requires. Lightweight learning at the edge, or TinyML, has made on-device prediction practical and energy-efficient, forecasting pollutant values on microcontrollers [22,23,24,25] and benchmarking across embedded platforms [26,27,28], and on-device fault detection has been demonstrated for low-power nodes [29]. These advances move computation onto the device, yet they learn a fixed mapping or a known fault signature and do not let the device reason, without a reference, about whether a channel measures the quantity it nominally reports. Forecasting a value or flagging a modeled fault and diagnosing an unmodelled inconsistency are different problems, and the latter calls for causal reasoning over the physical relations among channels rather than a fitted mapping. Connecting large language models to sensor streams supplies that reasoning [30,31,32,33], building on chain-of-thought and action-reasoning prompting [34,35] and the wider move toward autonomous agents [36,37], which is now reaching AIoT settings where LLM agents orchestrate device actions [38]. Yet these models run in the cloud, so the real-time sensor state on which any diagnosis depends must first traverse the network, and the capability disappears the instant the link does. Skill- and plug-in-driven approaches to sensing have likewise addressed neighboring problems, namely on-device program synthesis and zero-shot human activity recognition [39,40], rather than the runtime understanding and management of a new environmental sensor. Edge computing frames the alternative directly by pushing computation to the point where data are generated so that the device itself can perceive, reason, and act [41,42,43,44].

We pursue that alternative with Zhiwei, a personal-exposure monitoring system that internalizes the reasoning loop on the device. Built on a Raspberry Pi 5, Zhiwei runs an edge AI agent with reasoning, diagnosis, and memory. Its central, sensor-native contribution is reference-free on-device sensor-health self-diagnosis. By reasoning over the physical consistency among co-located channels, the device attributes apparent drift to its true cause, identifies cross-interference, and provides an on-device aging-trend indicator, all without a reference instrument or a network connection. Two mechanisms make this possible at deployment scale. A five-layer Sensor Intelligence Skill Package describes each sensor declaratively and, through a Capability Association Graph, lets the agent adopt a new sensor in seconds and compose cross-sensor analyses that no single channel supports. A three-tier reasoning architecture spanning a large cloud language model, a local lightweight model, and a rule engine then keeps quality control running as connectivity degrades, falling back to the device whenever the network is unavailable.

Table 1 places Zhiwei against representative prior systems along the capabilities that matter for autonomous quality assurance. No existing system simultaneously reasons on the device, monitors multiple pollutants, diagnoses its own sensors in real time, and manages new sensors at runtime; each compared system lacks at least one of these. To our knowledge, within this conjunction of capabilities Zhiwei is the first complete autonomous edge AI agent realized on multi-pollutant personal-exposure hardware, a claim we make over the specific intersection rather than over any one attribute alone.

Zhiwei’s contribution is the feasibility and architectural validation of on-device autonomous sensor quality assurance, not an improvement in the absolute metrological accuracy of low-cost sensors. The 30-day deployment used a single indoor site without a co-located reference-grade instrument, so the absolute concentrations and the exposure and infiltration metrics derived from them are reported as semi-quantitative and robust to order of magnitude, and their absolute calibration awaits a co-located reference, as discussed in Section 4.1. Within this boundary the manuscript reports the quantitative evidence it does have, summarized with sample sizes and uncertainties, and labels every result by its evidence level in Section 3.1. The remainder of the paper describes the system and its implementation in Section 2, reports the 30-day deployment results in Section 3, discusses limitations, generalizability, and privacy in Section 4, and concludes in Section 5.

## 2. Materials and Methods

This section presents the system on which the results of Section 3 rest. Section 2.1, Section 2.2, Section 2.3, Section 2.4, Section 2.5 and Section 2.6 describe the closed-loop agent architecture and its six subsystems; Section 2.7 describes the skill-package mechanism for runtime sensor management; and Section 2.8, Section 2.9, Section 2.10 describe the hardware, software, and deployment. The reference-free sensor-health self-diagnosis subsystem of Section 2.3 is the primary contribution, and the remaining components exist to enable and support it.

### 2.1. Overall Design Principles

**Figure 1:**
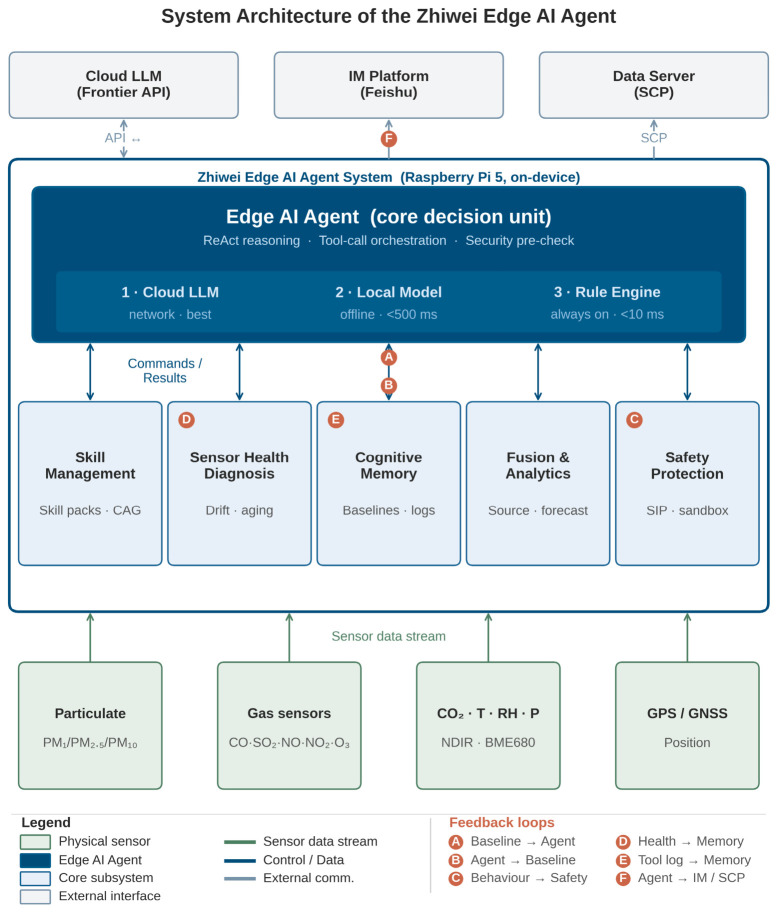
Overall architecture of Zhiwei. Six core subsystems form a closed-loop autonomous system through six feedback paths. The edge AI agent occupies the central position, providing output to and receiving input from the other five subsystems. Letters A–F mark the six feedback paths listed in Table 2. Double-headed arrows, including the cloud link marked “API”, denote bidirectional communication, and single-headed arrows one-way flow; line styles are defined in the legend inside the figure.

The design is organized around autonomy: under any network condition, the system should retain complete data-collection, quality-control, and anomaly diagnosis capability and run without human intervention. The architecture is therefore a closed loop in which six functional subsystems are coupled through six feedback paths, shown in Figure 1 and listed in Table 2. Where a conventional cloud pipeline moves data in one direction, from collection through upload to processing, these paths give the system a bidirectional, continuously self-correcting character. Paths A through E keep the internal state-update cycle intact even while the network is down, and path F resends backlogged alerts once the link returns.

### 2.2. Edge AI Agent and Three-Tier Resilient Reasoning

**Figure 2:**
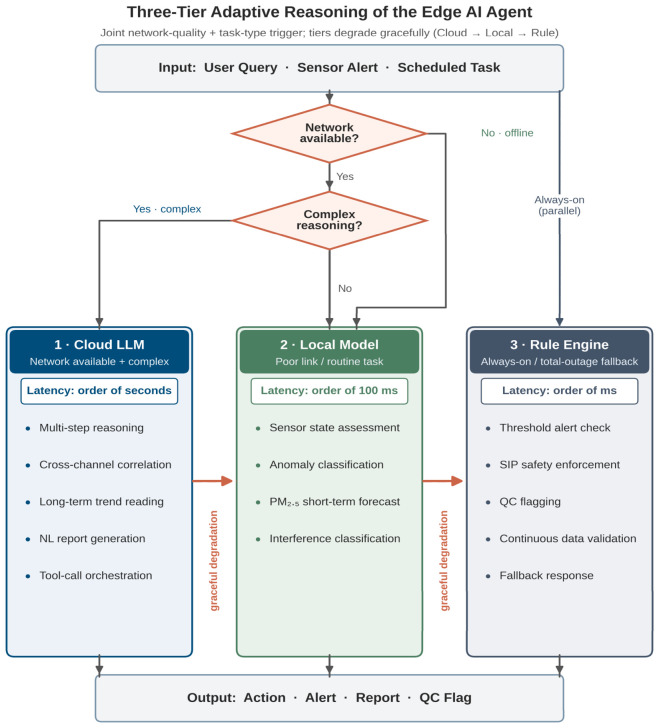
Adaptive switching among the three reasoning tiers. The system selects the tier from the network state and the task type so that a corresponding level of reasoning service remains available under any network condition.

The edge AI agent is the system’s decision core. Following the perceive–reason–act paradigm [44], it takes data queries from the sensor layer and natural-language instructions from messaging platforms, draws on several persistent memories, reasons, and emits either a response or a sequence of tool calls. Its reasoning is graded to the situation, as shown in Figure 2. When connectivity is good and the task is genuinely hard, such as interpreting cross-channel correlations, reading long-term trends, or writing a natural-language report, the cloud large-language-model tier carries it. A local lightweight model handles routine interpretation when the link is poor. A rule engine executes preset logic on the device when the task needs only a deterministic verdict, such as a threshold alert or a data-quality flag, or when the network has failed entirely. Because the tiers degrade into one another, a corresponding level of service survives any network condition.

Before every tool call, a two-factor pre-check screens the request: a hard rule layer rejects non-compliant instructions, and a behavioral layer flags calls that deviate from normal tool-use statistics, as detailed in Section 2.6.

### 2.3. Reference-Free Sensor-Health Self-Diagnosis

On the device and without a reference instrument, this subsystem detects the degradation modes it can check without a reference by accumulating evidence over repeated clean-night windows, and it grades each channel as trustworthy or not before that channel’s data enter any downstream analysis. The distinguishing idea is that diagnosis proceeds by physical-consistency reasoning across co-located sensors rather than by extrapolating a single channel’s trend, which is what allows a genuine fault to be told apart from an environmental confounder with no external ground truth.

During known pollution-free intervals, such as the low-activity hours of the late night, the system does not read a rising baseline as drift directly. It first regresses that baseline on the co-located confounders of temperature and humidity, restricting the fit to windows in which source-tracer channels such as CO, PM_2.5_, CO_2_, and VOC are simultaneously low, and declares a real drift only when the confidence interval of the residual trend, after these influences are removed, still excludes zero; otherwise, the trend is reported as inseparable from its confounders. This is what prevents an environmental confounder from being mistaken for a sensor fault, as the quantitative case of Section 3.2 shows.

Cross-interference is handled with the known interference structure of electrochemical cells. The channel that nominally reports ozone, for instance, has a near-unity theoretical cross-sensitivity to NO_2_, so the clean-night regression measures the field cross-interference slope and tests its sign against atmospheric chemistry, the analysis carried out in Section 3.2.

### 2.4. Environmental-Cognition Memory

Four persistent memories give the reasoning engine its long-term context: an environmental baseline memory of the site’s time-of-day and seasonal pollutant distribution, used in Section 3.6 to separate ordinary fluctuation from a genuine anomaly, together with a sensor-health memory of each channel’s diagnostic history, a dialog-analysis memory for cross-session context, and a safety-behavior baseline of normal tool calls.

### 2.5. Multi-Sensor Intelligent Fusion

The fusion subsystem carries out the cross-channel analysis that single channels cannot and is the layer at which composed capabilities execute. By reading the gas channels for CO, NO, NO_2_, SO_2_, O_3_, and VOC together with the particulate channels for PM_1_, PM_2.5_, PM_10_, and TSP and the meteorological channels for temperature, humidity, and pressure, it recognizes compound-pollution signatures, such as the fingerprint of a combustion source family, that no channel reveals alone. This capability underlies the source-apportionment and anomaly attribution results of Section 3.3, Section 3.4, Section 3.5 and Section 3.6.

### 2.6. Security Module

Because the agent can call tools in a networked environment, its execution is constrained at two stages by design. The two-factor pre-check of Section 2.2 screens each call with a hard-rule whitelist of permissible tool types and parameter ranges and a behavioral check against normal sliding-window statistics, and a secure-computing sandbox then bounds the system calls a process may make. Together these bound what the agent can do and block malicious or anomalous tool execution.

### 2.7. Skill-Package-Based Dynamic Sensor Management

Both self-diagnosis and fusion presume that the sensor is already understood: the system must know in advance each sensor’s physical meaning, data format, and quality behavior. The skill-package mechanism is what maintains that understanding dynamically, at runtime.

#### 2.7.1. Design Motivation

Sensor management must cross a semantic gap. At the hardware level a sensor emits digital signals over a serial, I2C, or Modbus interface, whereas the reasoning system must work in terms of meaning, for example, that CO sits within its normal range and that, read together with NO_2_, it shows no traffic-source signature. Zhiwei bridges that gap with the Sensor Intelligence Skill Package, which captures the knowledge needed in a declarative file that a loading engine parses and executes at runtime without touching the agent’s core program. We prefer a declarative description to generated code because dynamically loaded code is hard to audit on a resource-constrained device, whereas a declarative file carries no executable logic, is cheap to verify against a whitelist, and lends itself to version control and over-the-air update.

#### 2.7.2. Five-Layer Knowledge Encapsulation

**Figure 3:**
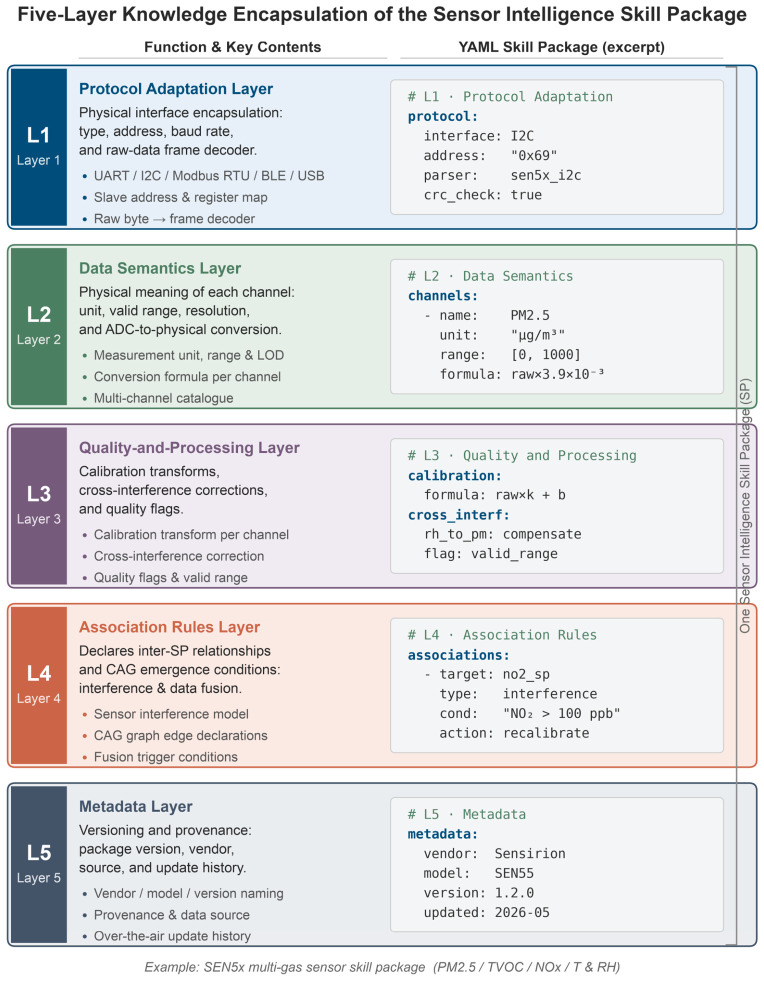
The five-layer knowledge encapsulation of the Sensor Intelligence Skill Package. Each layer resolves a distinct level of semantic conversion, and together they form the knowledge through which the agent understands and uses a sensor.

A skill package organizes a sensor’s knowledge into five layers, shown in Figure 3: a protocol-adaptation layer for the physical interface and raw-data parsing; a data-semantics layer fixing each channel’s unit, range, resolution, and detection limit; a quality-and-processing layer for calibration transforms, cross-interference corrections, and quality flags; an association-rule layer declaring cross-channel dependencies and the logic that activates a composed capability; and a metadata layer for versioning and provenance. Package files follow a vendor, model, and version naming convention supporting version management and over-the-air update.

#### 2.7.3. Skill-Loading Engine

**Figure 4:**
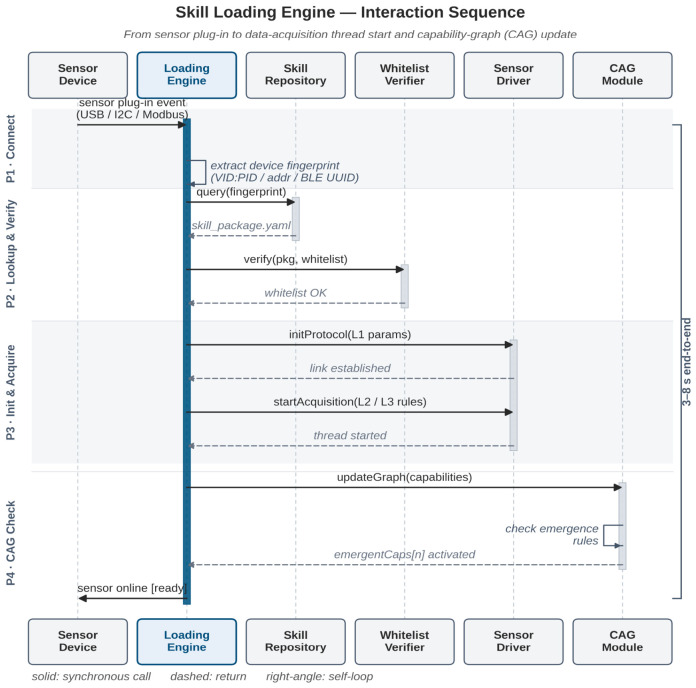
Interaction sequence of the skill-loading engine, from sensor connection through data-collection-thread startup to the capability-emergence check, completing end-to-end in three to eight seconds. Solid arrows denote synchronous calls, dashed arrows returns, and right-angle connectors self-loops.

The loading engine is the runtime core, shown in Figure 4. On connecting a sensor, it recognizes the device fingerprint, matches the corresponding skill package from the local library or a remote repository, parses and whitelist-verifies it, and starts the data-collection thread, completing the sequence in three to eight seconds, as reported in Section 3.7. A domain-specific language interpreter executes the association-rule layer under strict sandbox constraints, with read-only access to other channels, a 100 ms execution budget, and no network access, so that a third-party package can run safely.

#### 2.7.4. Capability-Emergence Mechanism

**Figure 5:**
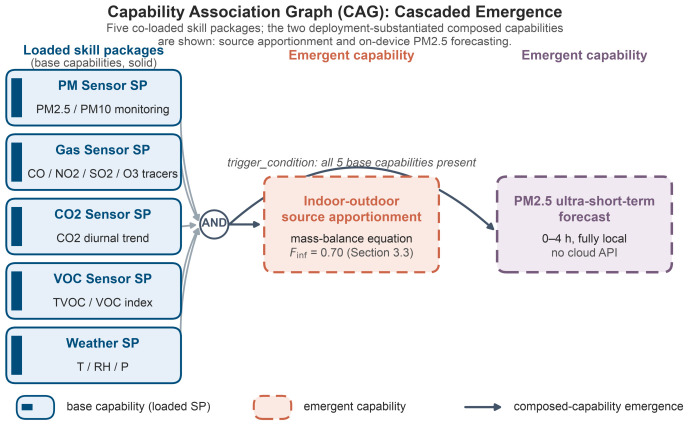
The Capability Association Graph and cascade activation. Solid nodes are basic capabilities from loaded packages, and dashed nodes are composed capabilities that emerge once their activation conditions are met; arrows indicate capability dependencies.

Composed capabilities are governed by the Capability Association Graph, a directed graph whose nodes are the basic capabilities that loaded packages provide, whose edges are dependency relations, and whose edge conditions state what must hold for a composed capability to activate, as illustrated in Figure 5. Loading a package triggers an emergence check that adds the new capabilities as nodes and then activates any composed capability whose dependencies are now all satisfied, adding it in turn and recursing so that emergence cascades through several levels; unloading reverses the process. Loading the particulate, gas, CO_2_, VOC, and meteorology packages together, for example, satisfies the dependencies of the indoor–outdoor source-apportionment rule and brings that capability into being, as realized in Section 3.3.

### 2.8. Hardware Platform

**Figure 6:**
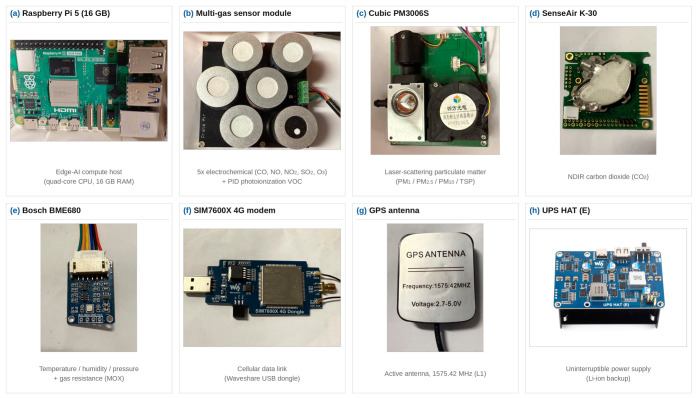
Physical modules of the Zhiwei hardware. (**a**) Raspberry Pi 5 edge AI host with 16 GB RAM; (**b**) six-in-one gas module with five electrochemical sensors for CO, NO, NO_2_, SO_2_, and O_3_ and one photoionization VOC sensor; (**c**) Cubic PM3006S laser-scattering particulate sensor; (**d**) SenseAir K-30 NDIR CO_2_ sensor; (**e**) Bosch BME680 temperature, humidity, and pressure sensor; (**f**) SIM7600 4G module; (**g**) GPS antenna; (**h**) uninterruptible-power module. All modules shown were used in the deployment.

Zhiwei is built on a Raspberry Pi 5 as a single edge-computing and acquisition core, shown in Figure 6 and specified in Table 3, which carries edge AI inference, concurrent multi-protocol sensor acquisition, and wireless data return on one platform. The sensor channels are mutually independent, so the failure of one does not propagate to the others.

### 2.9. Software Architecture and Automatic Operations

The software is written in Python 3.9 over a modular stack. A configuration layer governs sensor parameters, sampling, and communication; a sensor layer abstracts the heterogeneous protocols behind a factory-and-registry interface; a data layer runs one acquisition thread per channel and merges them through a thread-safe queue; a service layer provides local persistence and dual-mode transfer with resumable upload; and a core layer handles global scheduling, lifecycle management, and exception recovery. Every record carries a millisecond UTC timestamp, a WGS84 position, all channel readings, quality flags, and device-state metadata.

Unattended operation over long periods depends on a few mutually reinforcing safeguards. A systemd watchdog restarts the acquisition service and the agent within five seconds of any failure and brings them up automatically on boot. Charge monitoring on the uninterruptible-power module is read every five minutes and raises an alert below twenty percent so that data are saved and the device shuts down cleanly during a mains interruption. Disk monitoring and log rotation hold storage growth in check, with one raw-data file written per day and an alert when there is less than eight gigabytes of free space. A weekly health report summarizing per-channel completeness, anomaly counts, drift trends, and forecast-model error is pushed to the operator. The three delivery channels, instant message, email, and secure file transfer, are scheduled by priority while the network is available, cached while it is down, and resent on recovery.

### 2.10. Deployment Configuration

The device ran continuously at a fixed indoor location at the Institute of Atmospheric Physics, Chinese Academy of Sciences, in Beijing, from 7 May to 5 June 2026, spanning about 30 days or 710 h. The gas and CO_2_ sensors sampled indoor air by passive diffusion, and the particulate sensor drew air actively. Outdoor reference concentrations were taken, in order of preference, from the nearest national-control station, the Olympic Sports Center station 1007A, about 2 km away; then from a public aggregation platform; and finally from an early-morning low-activity baseline when online sources were unavailable. Sampling was continuous at about one record per channel every five seconds, stored locally and uploaded over 4G when a link was present. Because station 1007A lies about 2 km from the site, it serves as a comparison anchor for relative consistency and order-of-magnitude bias estimation rather than as a co-located reference-grade instrument, a distinction taken up in Section 4.1.

## 3. Results

This section reports Zhiwei’s measured results over a 30-day continuous indoor single-site deployment in Beijing. It asks whether the system runs reliably without human intervention, whether sensor self-diagnosis and skill emergence hold up in a real setting, and how far on-device reasoning can extend the analytical reach of personal-exposure monitoring.

### 3.1. Deployment Overview and Data Quality

Across the 30 days, the four environmental-sensor classes, namely the BME680 meteorological sensor, the Cubic particulate sensor, the K-30 CO_2_ sensor, and the six-in-one Modbus gas group, recorded 1,896,789 measurements at about one sample per channel every five seconds. Against the theoretical count of about 1,898,030 at that interval, raw completeness is about 99.9%. The channels were then aligned and resampled onto a gap-free one-minute grid of 42,581 timestamps spanning 7 May to 5 June 2026, on which every environmental channel is fully populated. Completeness throughout this paper means the number of records actually written by the four environmental-sensor classes divided by the theoretical number at the measured interval; the GPS channel, which has no satellite signal indoors, is excluded. Table 4 summarizes the deployment. Table 5 consolidates the quantitative results with their sample sizes and uncertainties, and Table 6 assigns each result its evidence level.

### 3.2. Reference-Free Self-Diagnosis of the Oxidizing-Gas Channel

**Figure 7:**
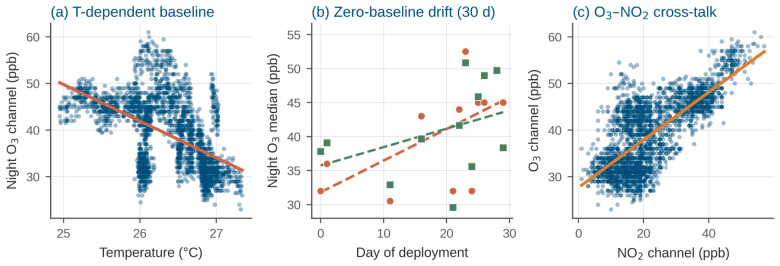
On-device self-diagnosis of the low-cost oxidizing-gas channel over 30 days of per-minute real data, with no reference instrument. Three complementary checks during clean late-night hours from 00:00 to 05:00 flag the channel as untrustworthy as an ozone reading. Panel (**a**) shows the temperature dependence of −7.9 ppb per °C with r = −0.53. Panel (**b**) shows that the apparent zero-drift of +0.46 ppb per day at *p* = 0.04 falls to a non-significant +0.27 ppb per day at *p* = 0.20 after temperature and humidity are removed, with the raw trend plotted in red and the corrected trend in green. Panel (**c**) shows the positive correlation with NO_2_ of +0.51 ppb per ppb with r = +0.73, the wrong sign for real ozone, identifying a combined ozone-and-NO_2_ response.

Before reporting any reading, a personal-exposure device must decide which of its own channels to believe. Lacking a reference instrument, the self-diagnosis subsystem instead tests each gas channel for internal physical consistency and grades its usability accordingly. For the channel that nominally reports ozone, the AlphaSense OX-B431, a nominal O_3_ electrochemical cell that by design responds to total oxidizing gas (O_3_ + NO_2_), it concludes that the reading cannot be taken as a real ozone concentration, and it reaches that verdict from three complementary lines of physical evidence, shown in Figure 7.

The first is thermal: across the clean hours of the late night, the reading tracks indoor temperature at about −7.9 ppb per °C, with r = −0.53, a dependence characteristic of the electrochemical cell’s intrinsic temperature sensitivity rather than of atmospheric ozone. That same temperature dependence then disposes of what would otherwise look like aging drift. Taken alone, the zero baseline rises at +0.46 ppb per day, a trend a static detector would flag, but once temperature and humidity are entered into the regression, the residual falls to a non-significant +0.27 ppb per day at *p* = 0.20, indicating that temperature and humidity account for a substantial part of the apparent trend, with no statistically significant residual drift remaining at this sample size. The third and decisive line is chemical: the channel correlates positively with NO_2_, at +0.51 ppb per ppb with r = +0.73, whereas genuine atmospheric ozone is titrated away by NO and must therefore correlate negatively; a positive sign is inconsistent with pure ozone and indicates that the channel measures total oxidizing gas rather than ozone alone, its reading strongly influenced by NO_2_.

This sensitivity to NO_2_ is an intrinsic property of this class of electrochemical oxidizing-gas cell, not a sign of damage; it is exactly what the manufacturer documents, which names the OX-B431 an “Oxidising Gas Sensor − Ozone + Nitrogen Dioxide” and recovers ozone only by subtracting an independent NO_2_ sensor (O_3_ = OX-B431 − NO2-B43F) [45]. With no such reference, the device rediscovers that cross-sensitivity on its own. The high nighttime reading that might otherwise look like excess ozone is the same effect: indoor NO_2_ accumulates overnight, and across the 24 h of the day medians, the two channels share one diurnal cycle (r = 0.88); the oxidizing-gas median over 00:00–05:00 is near 43 ppb against about 36 ppb by day, in step with NO_2_ near 20 ppb against about 12 ppb by day, whereas real ozone would fall at night (Figure S1). What matters is that the device, with no reference instrument, recognizes on its own that the channel’s nominal quantity, ozone, is inconsistent with the physical quantity it actually measures, and grades it as unfit for quantitative ozone use, a judgment a passive terminal cannot make at the point of collection. The same framework affirms the relative usability of two other channels: the PM_2.5_ channel correlates at 0.90 with the nearest national station (Section 3.3), and the NO_2_ channel is corroborated by the close synchrony of its diurnal cycle with an independent outdoor reference, at r = 0.86 (Section 3.6). For NO_2_, trustworthy refers to relative rhythm and direction of change rather than absolute concentration, and that relative consistency is what lets it serve as a cross-validation anchor while avoiding the circular trap of validating one uncalibrated channel against another. This attribution logic is the sensor-health skill-package method of Section 2.3.

### 3.3. Indoor–Outdoor Source Apportionment of PM_2.5_

A central question for personal-exposure monitoring is where the exposure comes from, because the same PM_2.5_ reading carries a different health meaning and calls for a different intervention depending on whether it originates indoors or infiltrates from outdoors. Zhiwei answers it on the device with a source-apportionment skill grounded in the indoor-pollutant mass-conservation equation [46,47]:

dC_in_/dt = P·a·C_out_ − (a + k)·C_in_ + S/V (1)

Here C_in_ and C_out_ denote the indoor and outdoor concentrations, P the penetration factor, a the air-exchange rate, k the indoor deposition and reaction loss rate, S the indoor source-emission rate, and V the indoor volume. At steady state, the outdoor-infiltration contribution is the product of an infiltration factor, F_inf_, and C_out_, where F_inf_ equals P·a divided by the sum of a and k, and the indoor-source contribution is S divided by the product of V and that same sum. The deployment estimates an effective infiltration factor, written F_inf_, that folds penetration, ventilation, and deposition loss into one equivalent coefficient.

**Figure 8:**
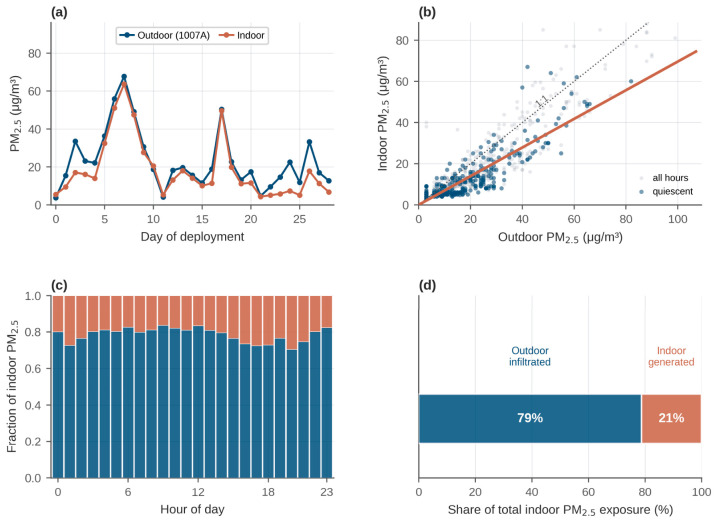
Indoor–outdoor PM_2.5_ source apportionment, comparing the Zhiwei indoor reading with the national station 1007A over 30 days of hourly real data from 7 May to 5 June 2026, with the outdoor source taken from the China National Environmental Monitoring Center through a third-party open-data archive. Panel (**a**) shows the indoor and outdoor PM_2.5_ time series with r = 0.90. Panel (**b**) shows the origin-forced regression during quiescent periods, giving an infiltration factor of 0.70 from 311 h, with the dotted line marking the one-to-one relation and points above it corresponding to indoor-generation events. Panel (**c**) shows the hourly apportionment into outdoor infiltration and indoor generation. Panel (**d**) shows the 30-day cumulative split of about 79% outdoor infiltration and 21% indoor generation, with a median indoor-to-outdoor ratio of 0.78.

The skill runs as a short chain of inferences. During quiescent periods, when the indoor combustion tracers CO and CO_2_ are simultaneously low, an origin-forced regression of indoor on outdoor PM_2.5_ gives a slope that is the effective infiltration factor, drawn here from 311 qualifying hours (Figure 8). With that factor in hand, each hour is divided into an outdoor-infiltration contribution and an indoor-generation contribution; peaks in the indoor-generation series are isolated by a dynamic threshold to mark indoor-generation events, and the apportionment is finally cross-checked against the indoor–outdoor correlation and against a combustion fingerprint of synchronous CO, SO_2_, and VOC rises so that no single coefficient is left to drive the conclusion. Outdoor reference concentrations come from station 1007A through the priority order of Section 2.10.

The indoor and outdoor PM_2.5_ hourly series move closely together, at r = 0.90 with a median indoor-to-outdoor ratio of 0.78, which on its own points to outdoor dominance. On the quiescent subset, the origin-forced slope gives an effective infiltration factor of 0.70, reported as indicative and semi-quantitative since the national station about 2 km away reflects the city-scale background rather than the building facade, a value physically reasonable for partial ventilation combined with deposition loss. Several checks support that estimate: an ordinary-least-squares fit with an intercept leaves that intercept non-significant at t = −0.8, day-block bootstrap resampling places the slope within about ±0.04, and the factor stays between 0.70 and 0.79 across different quiescence criteria. Over the 30-day cumulative exposure, outdoor infiltration accounts for about 79% and indoor generation for about 21%. The strong correlation and the infiltration-dominated split point the same way: PM_2.5_ exposure at this site was driven mainly by outdoor infiltration over the monitoring period, while indoor generation remained a secondary but real contribution whose sporadic spikes carry the combustion fingerprint examined in Section 3.5. The absolute calibration of the infiltration factor and of the split is bounded by the use of low-cost sensors and a non-co-located outdoor reference, as Section 4.1 discusses, but the qualitative conclusion does not rest on the precise value of the factor.

### 3.4. Safe-Autonomy Gating for On-Device Forecasting

On the Raspberry Pi 5, Zhiwei runs a fully local PM_2.5_ ultra-short-term forecasting skill over a zero-to-four-hour horizon at fifteen-minute steps, with no dependence on a cloud service. This result concerns safe autonomy rather than forecasting accuracy: it tests whether the device can recognize that its own model is not yet reliable, decline to put it into service, and fall back to a safe baseline so that over this deployment no forecast worse than the baseline reaches the user. Because no online forecasting service ran during the deployment, the result is an offline walk-forward replay over the real 30-day series.

**Figure 9:**
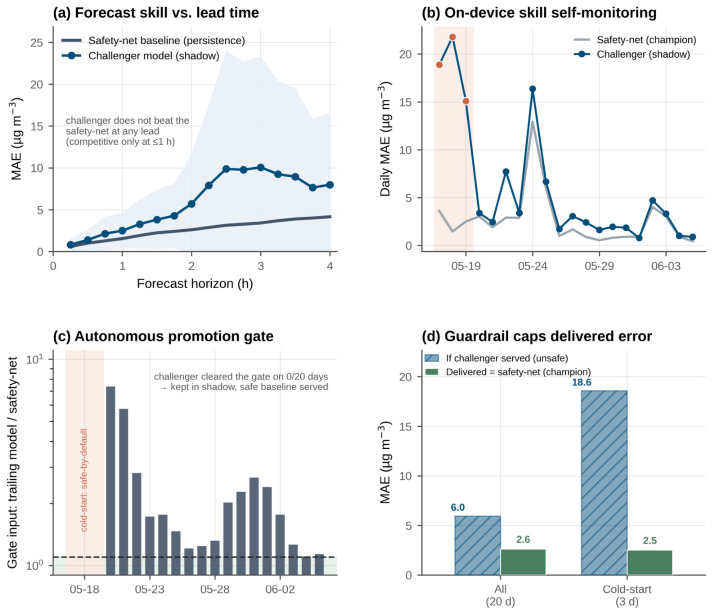
On-device champion–challenger gating for PM_2.5_ ultra-short-term forecasting over the 20 evaluation days within the 30-day series, evaluated by offline walk-forward replay. Panel (**a**) shows forecast skill against lead time, where the challenger with its one-sigma band never beats the persistence baseline. Panel (**b**) shows the daily same-day mean absolute error of the challenger and the baseline, with the first three cold-start days shaded. Panel (**c**) shows the daily causal promotion gate, whose rolling three-day error ratio stays above the 1.1 threshold on every day, so the challenger is promoted on none of the 20 days. Panel (**d**) shows that the gated fallback caps the delivered error, reducing the raw error of 5.95 over 20 days and 18.58 at cold start to 2.62 and 2.52 µg/m^3^.

The forecaster is a set of horizon-specific gradient-boosting models [48], one per target instant, each predicting directly from the current observation to avoid the error accumulation of recursive prediction [49]. It draws on the multi-size particulate readings; the BME680 temperature, humidity, and pressure; lag and rolling statistics over windows from fifteen minutes to twenty-four hours; and cyclically encoded time features. A full inference of the sixteen models on the Raspberry Pi 5 takes about 420 ms, occupies about 180 MB, and peaks near 40% CPU during the 420 ms burst, under 1% of a core when time-averaged at the fifteen-minute cadence, leaving concurrent acquisition and quality control undisturbed. The skill is wrapped in a champion–challenger loop in which persistence serves as the always-on safe baseline and the gradient-boosting model runs alongside it as a challenger in shadow mode; each day the device reviews the rolling three-day performance causally and promotes the challenger only when its error ratio drops below the promotion threshold of 1.1, which requires the challenger’s three-day error to come within ten percent of the safe baseline before it can take over, a margin that also damps needless switching when the challenger is only marginally noisy, and otherwise holds to the baseline (Figure 9).

On this clean, strongly autocorrelated indoor series the challenger never improves on persistence. Its mean absolute error is about 5.95 µg/m^3^ against the baseline’s 2.62, and over the first three days it over-predicts at the longest horizons, lifting the cold-start error to about 18.58 µg/m^3^. Feature importance explains why, rather than implicating poor tuning: recent particulate lags carry almost all of the model’s gain at every horizon; the PM_1_, PM_10_ and PM_2.5_ lag and rolling terms together are from 91 to 99 percent; and the short PM_1_/PM_10_ lags alone are the largest group, while meteorology reaches at most about 8 percent of gain, and the cyclic time terms contribute almost nothing (Figure S2). The one thing to exploit is short-term persistence, which the baseline already captures, so the model’s extra capacity adds variance, not skill; under the same causal protocol, a heavily regularized model still gives 5.40 µg/m^3^ and a linear AR(1) model 3.42, neither approaching persistence. The cold-start error is, in the same way, not a meteorological confounder but a symptom of data scarcity: it sits at the long horizons while the training window is still only days long, and it is gone by the fourth day. The gate turns this into safe behavior. Its rolling three-day error ratio never falls below 1.108, so the challenger is promoted on none of the twenty days; even a unit threshold would promote on none, and although it edges below persistence on a single isolated day, the rolling criterion rightly declines to act on one day. Delivered error is held at the baseline’s 2.62 overall and 2.52 at cold start, with no regression reaching the user. The contribution is this judgment of when to trust a model and when to withhold it; showing that the gate can also promote a model on a series with genuine signal is left to the future work of Section 4.1.

### 3.5. Personal Multi-Pollutant Exposure Burden

**Figure 10:**
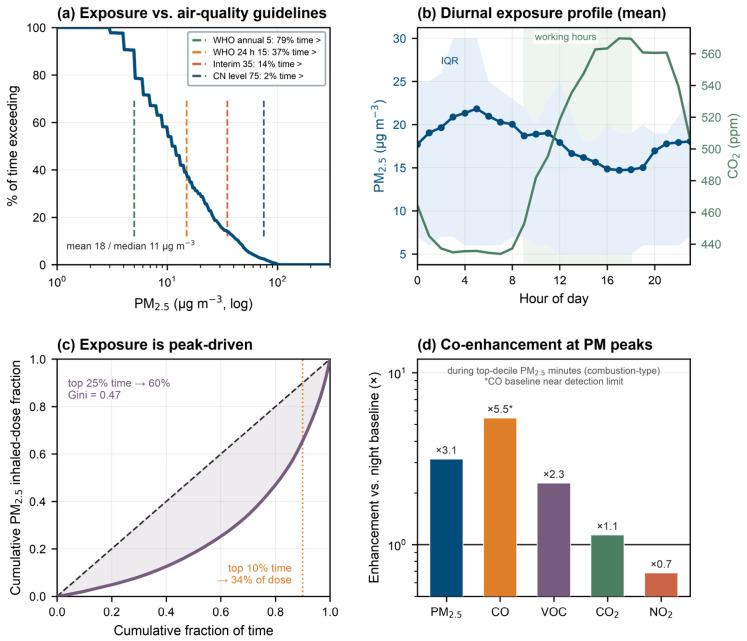
Personal multi-pollutant exposure assessment at a single indoor fixed location from 7 May to 5 June 2026, per minute, over 42,581 periods of real data. Panel (**a**) shows the PM_2.5_ complementary cumulative distribution on a logarithmic axis, with dashed lines marking the World Health Organization annual value of 5, the 24 h guideline of 15, the interim target of 35, and the Chinese Grade-II daily limit of 75 µg/m^3^, and exceedance-time fractions of about 79, 37, 14, and 2%; the time mean is about 18 and the median about 11 µg/m^3^. Panel (**b**) shows the PM_2.5_ mean diurnal variation with its interquartile band and the CO_2_ median in anti-phase. Panel (**c**) shows the Lorenz curve of inhaled dose against time, where the top 10% of time delivers about 34% of the dose, with a Gini coefficient of 0.47. Panel (**d**) shows the change in the top-decile periods relative to the nighttime baseline, with PM_2.5_ about 3.1 times, CO about 5.5 times (asterisk: CO baseline near the detection limit), VOC about 2.3 times, CO_2_ about 1.1 times, and NO_2_ about 0.7 times; the spikes carry a combustion-type fingerprint whose source is not identified.

The 30 days of monitoring yield the end-to-end exposure-burden assessment of Figure 10. The site PM_2.5_ averages about 18 µg/m^3^ with a median near 11 µg/m^3^, but the burden is far from evenly spread in time. Read against nominal thresholds, which are counting fractions rather than calibrated exceedances because the channel is not co-located-calibrated, about 37% of the time lies above the World Health Organization 24 h guideline of 15 µg/m^3^, about 14% above the interim target of 35 µg/m^3^, and about 2% above the Chinese Grade-II daily limit of 75 µg/m^3^. The mean concentration runs higher from night into early morning, near 20 to 22 µg/m^3^, and falls through the afternoon toward 15 µg/m^3^, in phase with the night-high NO_2_ rhythm and in anti-phase with the afternoon rise in CO_2_; because the hour-of-day median stays flat (Section 3.6), this night-high shape in the mean reflects a few episodic nighttime infiltration events lifting the mean above the median rather than a routine diurnal cycle, again pointing to outdoor infiltration rather than indoor activity as the driver.

The Lorenz curve makes the concentration explicit: the most-exposed tenth of the time delivers about 34% of the inhaled PM_2.5_ dose, a Gini coefficient of 0.47, so the burden is moderately concentrated, and a few short episodes contribute disproportionately. These are exactly the episodes that daily or monthly averaging would erase but that continuous high-frequency monitoring preserves. Set against the nighttime baseline, those top-decile periods show PM_2.5_ enhanced about 3.1 times alongside CO about 5.5 times (its baseline near the detection limit), VOC about 2.3 times, and CO_2_ about 1.1 times, while NO_2_ does not rise. Particulates and reducing or organic gases climbing together while NO_2_ declines slightly is the signature of a combustion source family, consistent with the indoor-generation events of Section 3.3, though the source itself is not identified. Dose-concentration measures such as the Lorenz curve and the Gini coefficient are robust to multiplicative calibration error, while the absolute concentrations remain bounded by the single fixed location and the uncalibrated low-cost channels, as Section 4.1 discusses.

### 3.6. Baseline Memory and Anomaly Detection

**Figure 11:**
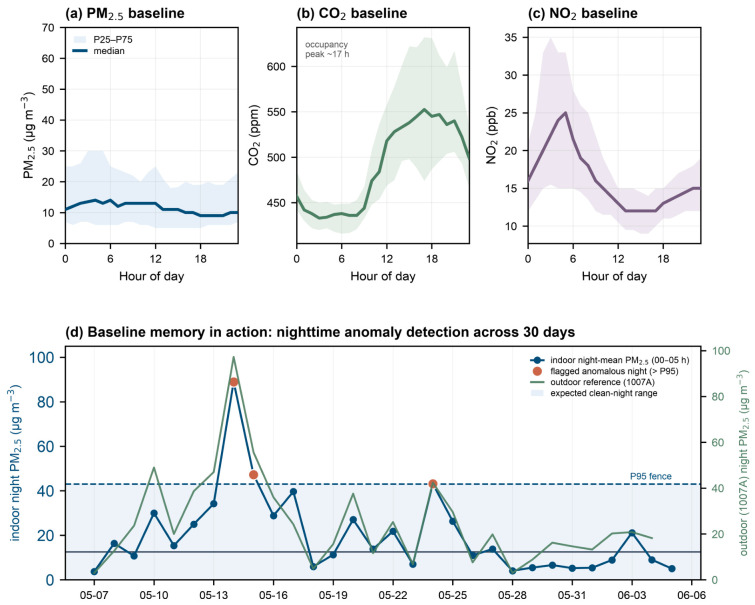
Construction and application of the environmental baseline memory over 30 days of per-minute real data. Panels (**a**–**c**) show hour-of-day baselines, with the solid line the per-hour median and the band the interquartile range: panel (**a**) a flat PM_2.5_ baseline near 9 to 14 µg/m^3^, panel (**b**) CO_2_ tracking occupancy, and panel (**c**) the relative-rhythm-validated NO_2_ channel with a morning-high, midday-low rhythm, the oxidizing-gas channel being excluded per Section 3.2. Panel (**d**) shows nighttime anomaly detection, comparing the 30-night indoor PM_2.5_ means from 00:00 to 05:00 with the learned baseline of median 12.5 (the solid line) and 95th percentile 43 µg/m^3^ (the dashed fence); the nights of 14, 15, and 24 May exceed the 95th percentile and are flagged, with station 1007A rising synchronously to about 97, 56, and 42 µg/m^3^.

The environmental baseline memory of Section 2.4 keeps the site’s time-of-day pollutant distribution current so that ordinary fluctuation can be separated from a genuine anomaly, as Figure 11 shows, holding for each channel an hour-of-day median and interquartile range. These baselines are themselves telling: the PM_2.5_ median baseline is essentially flat, with no routine diurnal peak (the night-high shape in the mean diurnal of Section 3.5 comes from a few episodic infiltration events rather than a nightly cycle), in keeping with the outdoor-infiltration dominance of Section 3.3; the CO_2_ baseline follows occupancy, near 435 ppm at night and peaking near 552 ppm around 17:00; and the relative-rhythm-validated NO_2_ channel traces a morning-high, midday-low rhythm, peaking near 25 ppb around 05:00 and dipping to about 12 ppb between 13:00 and 17:00, the shape expected from nighttime accumulation, morning-rush emission, and midday photochemical dilution.

Because the NO_2_ and oxidizing-gas channels share a device type, we checked whether the NO_2_ rhythm might itself be a thermal artifact. Against an independent outdoor reference, the national station is about 2 km away, and so for a city-scale rather than co-located comparison, indoor NO_2_ diurnal variation is closely synchronous over the 24 h of the day medians, at r = 0.86, both peaking in the early morning and troughing near 15:00, which indicates the channel is tracking a real atmospheric rhythm. Room temperature and atmospheric NO_2_ are strongly anti-correlated over the day, so the diurnal shape alone cannot fully separate the signal from any residual thermal response; it is the synchrony with the independent outdoor station that does the separating, which is why NO_2_ is used only as relative-rhythm evidence.

Anomaly detection then exploits the quiet late-night window from 00:00 to 05:00, over which the indoor PM_2.5_ nightly mean is tightly concentrated, with a baseline median near 12.5 and a 95th percentile near 43 µg/m^3^. Twenty-seven of the thirty nights fall within range; the nights of 14, 15, and 24 May are flagged for exceeding the 95th percentile, with nightly means near 88.9, 47.2, and 43.2 µg/m^3^, three and a half to seven times the baseline. Attribution across channels shows station 1007A rising in step to about 97, 56, and 42 µg/m^3^ on those nights, marking regional infiltration through doors and windows; because station 1007A lies about 2 km from the site and the indoor channel is uncalibrated, the indoor-to-outdoor magnitude ratio is not read here as a calibrated infiltration estimate. On 15 and 24 May the indoor CO tracer stayed near zero, identifying pure-infiltration anomalies, whereas on 14 May indoor CO rose in concert to a peak near 0.35 ppm, about four times its deployment-period 95th percentile, indicating a superimposed local combustion contribution whose source is not identified. The criterion is relative rather than absolute, so the same rule adapts on its own to a clean or a polluted site, triggering at whatever concentration is locally unusual.

### 3.7. Skill-Management Efficiency

To gauge the cost of adopting a new sensor, we set the time to onboard one under the traditional route of writing drivers, training a calibration model, and deploying the agent against Zhiwei’s skill-package route, summarized in Table 7. From sensor connection to a running data-collection thread, including the graph update, the loading engine takes three to eight seconds, measured over fifteen cold starts across the serial, I2C, and Modbus interfaces on the Raspberry Pi 5, where the traditional route takes two to seven working days.

Loading five skill-package classes together, covering particulates, the gases CO, SO_2_, NO, O_3_, and NO_2_, CO_2_, VOC, and meteorology, let the graph activate composed cross-sensor analyses, the demonstrated case being the indoor–outdoor source apportionment of Section 3.3. This configuration exposes 21 basic capabilities, from which 13 composed capabilities emerged, an illustrative ratio of 62%. This ratio is configuration-dependent, as Table 6 notes.

### 3.8. System Reliability and Unattended Operation

Over the deployment, the four environmental-sensor classes collected without interruption at about one sample per channel every five seconds, at about 99.9% raw completeness, and after resampling onto the one-minute grid, all 42,581 timestamps are valid with no gaps, giving every analysis above an unbroken substrate. The unattended operation behind this record was carried out by the systemd watchdog, automatic log rotation, the weekly health diagnosis, and uninterruptible-power charge monitoring of Section 2.9.

When the network or the agent service falters, the system drops from the cloud tier to the on-device tiers, first the local lightweight model and then the rule engine as the deterministic floor when the network fails entirely, and goes on acquiring and quality-checking data on the device, losing nothing once the link returns. No prolonged real disconnection occurred during these 30 days, so we measured the takeover directly with a controlled fault-injection test on the deployed Raspberry Pi 5: pausing the upstream proxy to simulate a cloud outage, the agent fell back to the on-device model in 5.1 s, and that model absorbed the inference load, its CPU rising to a 356% peak of the four-core SoC at about 3.9 GB resident, while the always-on collection process stayed flat and its on-disk records grew strictly monotonically with zero loss, gaining 51 records during the 90 s outage (Figure S3; Table 6). The measured takeover exercises the cloud-to-local-model tier; the rule-engine floor for total network failure remains design-validated. This is a controlled bench reproduction rather than a field cellular outage, and the contrast with a passive-terminal architecture that loses quality control during an outage remains an architectural argument rather than a head-to-head experiment against a co-located control device, a limitation taken up in Section 4.1.

## 4. Discussion

### 4.1. Limitations

The conclusions rest on a single device at a single indoor site over 30 days, with neither a co-located reference-grade instrument nor a co-located control device, and each should be read within that scope. The most consequential limit is that no claim of absolute accuracy is made: every channel is a low-cost sensor, no co-located reference was available, and the absolute concentrations and the exposure and infiltration metrics built on them are therefore semi-quantitative and robust only to order of magnitude, with the cross-station correlations of 0.90 for PM_2.5_ and 0.86 for NO_2_ serving as temporal-consistency checks against a reference about 2 km away rather than as accuracy validation. The r = 0.88 synchrony between the oxidizing gas and NO_2_ channels may itself carry some shared electronic or thermal noise, as the two cells share a module, though the independent outdoor corroboration of the NO_2_ channel at r = 0.86 substantially mitigates it. Absolute calibration awaits a co-located reference and a gravimetric comparison.

The remaining limits follow from the same single-deployment scope, and each narrows a specific claim rather than the architecture as a whole. One Beijing indoor site over 30 days cannot speak to multi-site behavior or to adaptability across outdoor and mobile environments, and a fixed indoor location is only a near proxy for true personal exposure along a trajectory. The forecasting result is an offline walk-forward replay rather than a live service, and on a clean, strongly autocorrelated series, the challenger did not beat persistence, so it demonstrates safe autonomy rather than forecasting accuracy; showing the gate both reject and promote will require a series with a genuine predictive signal. No prolonged disconnection occurred during the deployment, so resilient takeover is demonstrated by the controlled bench fault injection of Section 3.8 rather than by recovery statistics from a real field outage, and the comparison with a passive-terminal architecture is an architectural argument rather than a controlled experiment against a co-located device. A cross-interference correction transform and an aging-trend monitor, both declared in the skill package, run at deployment but were not separately field-validated; the NO_2_ cross-interference could in principle be removed in hardware by a heated catalytic ozone scrubber or a differential dual-electrode design [17], and the manufacturer’s reference method is itself such a subtraction (O_3_ = OX-B431 − NO2-B43F), already provisioned by the on-board NO2-B43F though unused here, but doing so would remove the very fault the device is meant to catch, so it is left, with a co-located reference, to future work. The capability-emergence ratio is illustrative and configuration-dependent, as Table 6 records. The local lightweight model is bounded by the compute of the Raspberry Pi 5, so demanding reasoning still benefits from the cloud tier, and a per-tier latency and throughput benchmark of the three reasoning tiers is reserved for a dedicated controlled experiment. Concurrency across skill packages is bounded in the same way: each additional heavy model costs about 180 MB and about 40% of a core during its inference burst, so the number of complex models running simultaneously is limited by cores, memory, and duty cycle, with staggered scheduling, model quantization or distillation, and cloud offload as the mitigations. Skill-package quality, finally, depends on the developer, and the review mechanism for third-party packages needs strengthening, as does a quantitative evaluation of the agent’s two-stage security layer, which the present deployment specifies by design but does not benchmark. None of these undermines the architectural feasibility the paper demonstrates; together they set the agenda for the work of Section 5.

### 4.2. Generalizability

The design carries over beyond this deployment with little adaptation. Its single-board-computer core ports to other ARM platforms, and its modular software and skill-package mechanism are independent of any particular sensor model, having been exercised across serial, Modbus, Bluetooth, and over-the-air interfaces. The governing principle, that perceptual intelligence belongs on the device, extends well past environmental monitoring. The source-apportionment and forecasting skills show further that a skill package can declaratively encapsulate not only sensor onboarding but also a physical model and a machine-learning model alike, a pattern of direct use for edge analysis in industrial monitoring and precision agriculture. The accompanying operations stack, with its process watchdog, power monitoring, disk warning, weekly health report, and remote file return, amounts to a lightweight remote-operations layer for the unattended deployment of low-cost sensors at a per-unit cost on the order of one to two thousand yuan [50].

### 4.3. Privacy and Security

Personal-exposure data can encode movement and therefore touch user privacy. Zhiwei limits this exposure through partitioned local storage with geo-fence-configurable desensitization of trajectory data, encrypted transport with mutual authentication, and the two-layer constraint on the agent’s tool calls described in Section 2.6. The present deployment tracked no person at all, since the device sat at a fixed indoor location, acquired no satellite fix, and recorded only ambient environmental variables, so no human-subjects data arose. Any large-scale research deployment should nonetheless operate within an informed-consent framework consistent with the applicable data-protection regulations.

## 5. Conclusions

Zhiwei makes the case that the perceptual intelligence of a personal-exposure device belongs on the device rather than on a remote server and that locating it there is what lets the device keep working under any network condition. The argument is carried by a single 30-day deployment of 1,896,789 high-frequency records at a Beijing indoor site, with every claim scoped to the evidence assembled in Table 5 and Table 6.

The central finding is that reference-free self-diagnosis can be performed on the device itself. With no reference instrument, reasoning over the physical consistency among co-located channels let the device grade the oxidizing-gas channel as untrustworthy for ozone: its apparent zero-drift lost significance once confounders were removed, falling from +0.46 to +0.27 ppb per day, while a strong temperature dependence of −7.9 ppb per °C and a sign-wrong correlation with NO_2_ of +0.51 ppb per ppb exposed it as an oxidizing-gas sensor, whereupon the system fell back to the consistency-validated NO_2_ channel. Judging a channel’s trustworthiness before its data are used is the point-of-collection quality control that a passive terminal cannot perform.

Around this finding, the enabling mechanisms held up in deployment. The declarative skill package and its capability-emergence graph cut sensor onboarding from days to seconds and exposed twenty-one basic capabilities, from which thirteen composed capabilities emerged in this single, illustrative configuration; one of them (the indoor–outdoor source apportionment of Section 3.3) demonstrated end-to-end, carrying the device from passive collection to quantitative exposure analysis. The three-tier architecture keeps quality control independent of the network by retreating to the on-device tiers, the local lightweight model backed by the rule engine as its deterministic floor, when the cloud or agent service is unavailable; the takeover is now established by design and by a measured bench fault-injection test (5.1 s fallback, zero record loss). Over the month, the deployment sustained roughly 99.9% raw completeness on a gap-free one-minute grid. The operations stack kept the device running unattended throughout and offers a reusable basis for large unattended networks.

Positioned between static edge prediction, which forecasts a value but cannot diagnose the cause of an anomaly, and cloud large-model analysis, which lapses when the link is lost, Zhiwei offers a device-autonomous, locally reasoning reference architecture for personal exposure monitoring. Its contribution is the feasibility and architectural validation of on-device autonomous quality assurance rather than a gain in the metrological accuracy of low-cost sensors. The natural next steps are to add a co-located reference instrument and a co-located control device for absolute calibration and a controlled resilience comparison; to network several devices with cross-device consistency checks for region-scale exposure cohorts [4,5]; to deploy quantized lightweight reasoning models tuned to the Raspberry Pi 5 and benchmark each reasoning tier; and to test the architecture under the volatile mobile conditions and rapid microenvironmental transitions and the high-density rural settings that a fixed indoor deployment cannot probe.

## 6. Patents

Two Chinese invention-patent applications arising from this work have been filed and are pending: “A Sensor Intelligence Skill Management System and Method for Edge AI Agents” (Application No. CN202610502779.X) and “A Multi-Source Heterogeneous Sensor Environmental Monitoring System and Method Based on an Edge AI Agent” (Application No. CN202610503452.4). The applicant is the Institute of Atmospheric Physics, Chinese Academy of Sciences, with Y. Wang as inventor.

## Figures and Tables

**Table 1:** Capability comparison of Zhiwei with representative prior systems. “Cloud” denotes a system whose reasoning runs on a remote server; “Prediction” a static on-device prediction model only; “Code” a capability confined to program generation; and “Partial” or “Limited” a capability present only within a constrained scope. N/A marks an attribute not applicable to that system’s task.

System	On-Device Reasoning	Multi-Pollutant	Real-Time Self-Diagnosis	Dynamic Sensor Management	Long-Term Deployment
SEARCH monitor [6]	No	Yes	No	No	Yes
AirPen [7]	No	Partial	No	No	Yes
TinyML air quality [22,24]	Prediction	No	No	No	Limited
IoT-LLM [30]	Cloud	N/A	No	No	No
LLMSense [32]	Edge + cloud	N/A	No	No	No
AQ-MCP interface [33]	Cloud	Yes	No	No	No
SensorLLM [39]	Cloud	N/A	No	Limited	No
AutoIOT [40]	Code synthesis	N/A	No	No	No
Zhiwei (this work)	Yes	Yes	Yes	Yes	Yes

Notes: The “Limited” entry for SensorLLM refers to per-channel zero-shot onboarding of inertial sensors only; AutoIOT operates in the program-generation domain and produces code rather than a runtime reasoning agent; the AQ-MCP interface [33] is multi-pollutant but runs its model in the cloud. “Long-term deployment” marks a reported continuous field deployment of at least several days.

**Table 2:** The six feedback paths of the closed-loop architecture.

Path	Information Flow	Function
A	Environmental baseline memory to edge AI agent	Baseline history aids anomaly judgment of the current observation
B	Edge AI agent to environmental baseline memory	The current observation continuously updates the baseline
C	Safety-behavior baseline to security module	Behavior patterns update the security-detection baseline
D	Sensor-health self-diagnosis to sensor-health memory	Diagnostic results are written back for long-term trend tracking
E	Tool-execution results to dialog-analysis memory	Execution history supports cross-session context consistency
F	Edge AI agent to messaging, email, and file transfer	Alerts, weekly reports, and data files are pushed and resent after recovery

**Table 3:** Hardware configuration of Zhiwei.

Component	Model and Specification	Main Function
Compute core	Raspberry Pi 5; BCM2712 quad-core Cortex-A76 at 2.4 GHz; 16 GB LPDDR4X	Edge AI agent, concurrent acquisition, processing, and communication
Particulate sensor	Cubic PM3006S laser scattering	PM_1_, PM_2.5_, PM_10_, and TSP plus six-channel particle-number concentration
Gas-sensor group	Six-in-one Modbus module integrating AlphaSense four-electrode (B4) cells: CO-B4, NO-B4, NO_2_-B43F, SO_2_-B4, and O_X_-B431 (oxidizing: O_3_ + NO_2_); plus an AlphaSense PID-AH photoionization VOC cell. Electrochemical channels at ±3%	CO, NO, NO_2_, SO_2_, and O_3_ electrochemical channels at ±3% plus a VOC channel
CO_2_ sensor	SenseAir K-30 NDIR, 0 to 5000 ppm, ±30 ppm	CO_2_ concentration
Meteorological sensor	Bosch BME680 over I2C	Temperature at ±1 °C, humidity at ±3%, pressure at ±1 hPa
Positioning and communication	SIM7600G-H GNSS and 4G LTE Cat-4	Positioning to 2.5 m and data return
Storage	64 GB microSD	Local persistence supporting more than 180 days of retention
Power	20,000 mAh lithium-polymer with UPS module	About 8 to 12 h of endurance under typical operation

**Table 4:** Overall deployment-result statistics.

Metric	Value
Deployment location	Institute of Atmospheric Physics, Beijing; fixed indoor location
Deployment period	7 May to 5 June 2026; about 30 days or 710 h
Total records collected	1,896,789 at about one sample per channel every five seconds
Raw-record completeness	About 99.9%, with GPS excluded
Unified one-minute grid	42,581 timestamps; all environmental channels fully available, no gaps
GPS	No satellite signal indoors; excluded from environmental-quality assessment
Sensor channels	11 pollutant parameters plus meteorological parameters
On-device autonomous analysis	Channel quality control, source apportionment, ultra-short-term forecasting, exposure-burden quantification, and baseline anomaly detection

**Table 5:** Consolidated quantitative results with sample sizes and uncertainty. All values come from real deployment data. Station 1007A is a non-co-located reference about 2 km from the site, so cross-station correlations are temporal-consistency checks rather than accuracy validation.

Quantity	Value	Sample and Uncertainty	Interpretation
PM_2.5_ indoor versus station 1007A	r = 0.90	Hourly pairs over 30 days; reference 2 km away	Indoor–outdoor temporal coupling, not accuracy
NO_2_ indoor versus station 1007A	r = 0.86	Hourly median diurnal cycle; *n* = 24 h-of-day medians	Relative-rhythm validation of the NO_2_ channel
Oxidizing-gas channel temperature dependence	−7.9 ppb per °C; r = −0.53	Clean nights, 00:00 to 05:00	Intrinsic thermal sensitivity of the cell, not damage
Oxidizing-gas channel cross-interference with NO_2_	+0.51 ppb per ppb; r = +0.73	Clean nights	Sign wrong for true ozone; an oxidizing-gas sensor
Oxidizing-gas channel apparent drift	+0.46 ppb per day at *p* = 0.04; +0.27 ppb per day at *p* = 0.20 after adjustment	Before and after confounder removal	No statistically significant real drift
Effective infiltration factor	F_inf_ = 0.70; range 0.70 to 0.79	311 quiescent hours; bootstrap 95% interval about ±0.04; intercept t = −0.8	Semi-quantitative; merges ventilation and deposition
Indoor–outdoor PM_2.5_ split	About 79% outdoor, 21% indoor	30-day cumulative	Order-of-magnitude robust, outdoor-dominant
Forecast challenger error	MAE = 5.95 µg/m^3^	20 evaluation days, walk-forward replay	Worse than baseline; not promoted
Forecast persistence baseline error	MAE = 2.62 µg/m^3^	20 evaluation days	Always-on safe baseline
Gated delivered error	2.62 over 20 days; 2.52 at cold start	Promoted on 0 of 20 days	Safe autonomy; no regression reaches the user
On-device forecaster cost	About 420 ms, 180 MB, and 40% CPU peak	16-model inference on the Raspberry Pi 5	Measured edge-resource cost
Exposure concentration	Gini 0.47; top 10% of time gives 34% of dose	42,581 one-minute periods	Robust to multiplicative calibration error
Sensor onboarding time	Three to eight seconds	15 cold starts across serial, I2C, and Modbus	Compared with two to seven working days traditionally

**Table 6:** Evidence level of each reported result. “Measured” denotes a quantity obtained directly from real deployment data or, for the row so marked, from bench fault-injection telemetry on the deployed device; “offline replay” denotes behavior reproduced by walk-forward replay on real data, with no online service running; and “design validation” denotes a capability implemented and verified by design but not separately field-validated in this deployment.

Result	Evidence Level	Basis
30-day collection, 99.9% completeness, gap-free one-minute grid	Measured	Real deployment
Oxidizing-gas channel graded untrustworthy (Section 3.2)	Measured	Multi-sensor internal consistency over 30 days
PM_2.5_ and NO_2_ channels confirmed usable in relative terms	Measured	Comparison with non-co-located station 1007A
Infiltration factor and outdoor–indoor split (Section 3.3)	Measured, semi-quantitative	Real data with a non-co-located outdoor reference
Forecasting gating behavior (Section 3.4)	Offline replay	Walk-forward replay; no online service ran
Exposure-burden quantification (Section 3.5)	Measured, semi-quantitative	Real data at a single fixed location
Baseline memory and anomaly detection (Section 3.6)	Measured	Real data with outdoor reference and CO tracer
On-device forecaster resource cost	Measured	Measured on the Raspberry Pi 5
Sensor onboarding of three to eight seconds (Section 3.7)	Measured	15 cold starts
Capability-emergence ratio of 62%	Design validation, illustrative	Single configuration; counting-criterion dependent
Resilient takeover and offline quality-control continuity	Measured (bench fault injection)	Bench fault injection on the deployed Pi 5: 5.1 s takeover, zero record loss, per-process CPU telemetry and record continuity (Figure S3)
Cross-interference correction and aging monitor	Design validation, not field-validated	Declared in the skill package; effect not measured here

**Table 7:** Sensor-onboarding cost: traditional development compared with the Zhiwei skill package.

Onboarding Mode	Typical Time	Modify Agent Core	Redeploy
Traditional development	Two to seven working days	Yes	Yes
Zhiwei skill package	Three to eight seconds over 15 trials	No	No

## Data Availability

The data supporting the findings of this study are available from the corresponding authors upon reasonable request.

## Supplementary Materials

The following supporting information can be downloaded at https://www.mdpi.com/article/10.3390/s26144526/s1, Figure S1: Hour-of-day medians of the oxidizing-gas (OX-B431) and NO_2_ channels over the 30-day deployment; Figure S2: Feature importance and ablation of the on-device PM_2.5_ forecaster from a causal walk-forward backtest; Figure S3: Measured graceful degradation under a controlled cloud-outage fault injection on the deployed Raspberry Pi 5.
