# Supplementary material for: An On-Device Edge AI Agent for Reference-Free Self-Diagnosis of Low-Cost Multi-Pollutant Sensors
Source: Sensors (Basel). 2026 Jul 16;26(14):4526. doi: 10.3390/s26144526 (PMC13418726; doi:10.3390/s26144526)
Supplement: Supplementary file 1 [file sensors-26-04526-s001.zip › sensors-4397395-supplementary.pdf]

Supplementary Materials

An On-Device Edge-AI Agent for Reference-Free Self-Diagnosis of Low-Cost Multi-Pollutant Sensors

Yinan Wang, Tianqi Wang, Yubing Pan

This file contains three supporting figures (Figure S1–S3) that provide the evidence requested by the reviewers. They are referenced from the main text (Sections 3.2, 3.4 and 3.8) and are reproduced here in full.

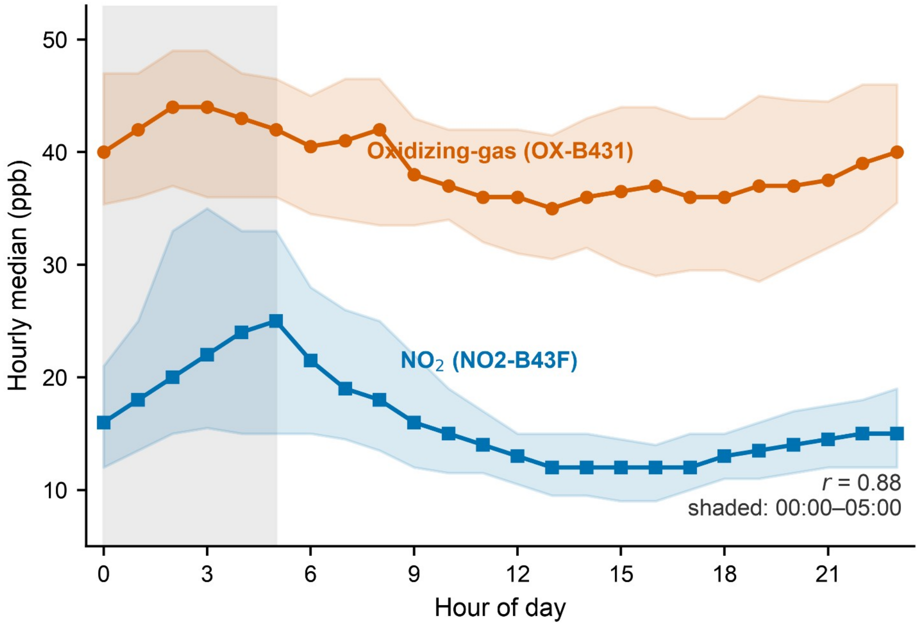

Figure S1. Reference-free corroboration that the oxidizing-gas channel tracks overnight NO_2 rather than ozone (Reviewer 1, comment 4). Hour-of-day medians over the 30-day deployment for the oxidizing-gas channel (AlphaSense OX-B431, orange) and the NO_2 channel (NO2-B43F, blue); shaded bands are the interquartile range of the one-minute readings at each hour of day, and the grey panel marks the 00:00–05:00 diagnostic window. The two channels follow the same diurnal cycle (Pearson r = 0.88), both rising at night, so the elevated nighttime oxidizing-gas reading reflects the O_x cell responding to overnight NO_2 accumulation rather than real ozone (which would instead fall at night through NO titration, deposition, and the absence of photochemistry).

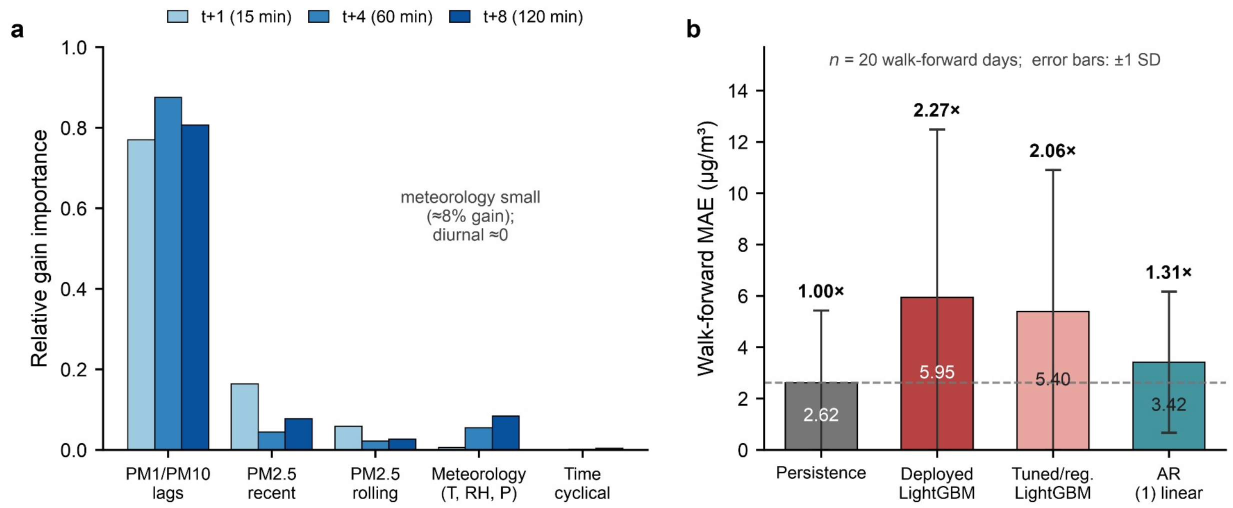

Figure S2. Feature importance and ablation of the on-device $PM_{2.5}$ forecaster, from a strictly causal walk-forward backtest (Reviewer 2, Major comment 2). (a) Relative gain importance by feature group at three forecast horizons (t+1, t+4, t+8): the PM_1/PM_{10}/$PM_{2.5}$ lag and rolling terms dominate (91–99% of gain per horizon, the short PM_1/PM_{10} lags alone the largest group), whereas meteorology contributes at most ≈8% of gain and the diurnal terms almost nothing. (b) Walk-forward mean absolute error (MAE) of persistence versus the deployed LightGBM, a heavily regularised/simplified LightGBM, and an AR(1) linear model (n = 20 walk-forward days; error bars are ±1 SD across days, clipped at zero since MAE ≥ 0; dashed line = persistence baseline; labels give each MAE in μg/m³ and its ratio to persistence). No learned model beats persistence, confirming that the loss is intrinsic to this short, stationary indoor series rather than a sign of under-optimisation.

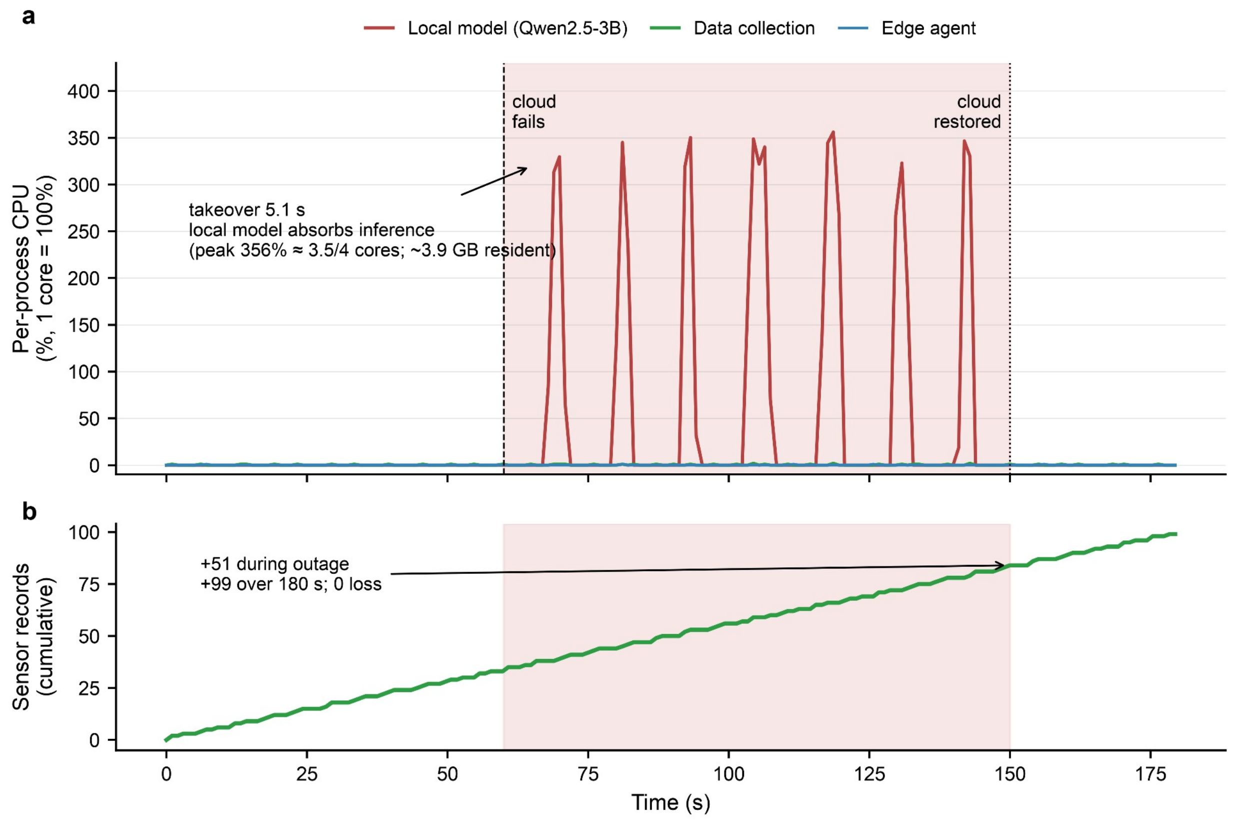

Figure S3. Measured graceful degradation under a controlled cloud-outage fault injection on the deployed Raspberry Pi 5 (Reviewer 2, Major comment 4). The shaded band marks the simulated outage (the upstream proxy was paused, t = 60–150 s). (a) Per-process CPU (1 core = 100%): on cloud failure the agent falls back to the on-device model in 5.1 s and the local model (Qwen2.5-3B) absorbs the inference load (peak 356%, ≈3.5 of the 4 cores, at ≈3.9 GB resident), while the edge-agent and data-collection processes remain flat. (b) Cumulative sensor records: data collection continues strictly monotonically across the outage with zero record loss (+51 records during the 90 s outage; +99 over the full 180 s telemetry window). This is a controlled bench reproduction of a cloud outage, not a field cellular outage.
